# Functional Bond-Selective Microscopy: Physically Informed Insights in Chemistry and Biology

**DOI:** 10.1021/acs.accounts.6c00380

**Published:** 2026-09-11

**Authors:** Philip A. Kocheril, Ryan E. Leighton, Noor Naji, Dongkwan Lee, Lu Wei

**Affiliations:** Division of Chemistry and Chemical Engineering, 6469 California Institute of Technology, Pasadena, California 91125, United States

## Abstract

Biological systems are immensely complex, comprising diverse molecular species organized across multiple spatial and temporal scales. Through intricate networks of tightly regulated chemical reactions, cells are able to maintain homeostasis, respond to environmental stimuli, and meet energetic demands. Despite massive technological advances spanning many decades, a significant gap still exists in our understanding of how biochemical networks are locally regulated in living systems. Fundamental physicochemical parameters such as concentration, pH, temperature, and electrostatics, which ultimately govern chemical reactivity, remain challenging to image quantitatively, particularly in live cells. We argue that developing methods capable of quantitatively imaging these physicochemical parameters is not only essential for interrogating the regulation of biochemical reaction networks but also provides a rich framework for uncovering hidden, physically informed insights in chemistry and biology.

To image chemical reactions in a biological context is a highly demanding task; few methods exhibit the necessary biocompatibility, spatial resolution, sensitivity, molecular specificity, and quantitative reporting capacity to access subcellular chemical information. Among existing optical methods, vibrational spectroscopy provides a natural context for understanding chemical reactions in cells, since molecular vibrations serve as chemical “fingerprints”, reporting quantitatively on the formation, breaking, and interactions of chemical bonds. Despite this enormous potential, conventional vibrational imaging faces significant challenges in quantifying chemical kinetics and dynamics in cells. Endogenous biomolecules exhibit highly overlapped, convoluted spectra, largely prohibiting specific molecular identification. Moreover, conventional techniques, generally based on infrared (IR) absorption or Raman scattering, have limited sensitivity, and in the case of IR absorption, coarse spatial resolution while operating at the diffraction limit, obscuring subcellular detail. As such, there is a significant need for vibrational spectro-microscopies with improved molecular specificity, sensitivity, and spatial resolution.

Coupling novel nonlinear optical strategies, which provide higher sensitivity and spatial resolution, with engineered vibrational probes, which afford higher molecular specificity and functionality, has proven fruitful in addressing these longstanding challenges in vibrational imaging and enabling functional bond-selective imaging. In this Account, we highlight recent work from our group toward quantitative measurements of physicochemical observables in biological systems. We first demonstrate the utility of small vibrational probes (deuterium labeling and alkyne tagging) with stimulated Raman scattering (SRS) microscopy for absolute concentration determination in protein aggregates and local pH sensing via hydrogen–deuterium exchange. We then discuss the development of two new imaging modalities, termed Boltzmann-edge vibrational thermometry (BET) and bond-selective fluorescence-detected IR-excited (BonFIRE) spectro-microscopy, and we highlight the applications of these methods toward quantitative measurements of local temperature and local electric fields. In each case, the pursuit of quantitative, physics-informed environmental reporting served as a launching point for the discovery of new biological or chemical phenomena. We believe that this is a valuable, unifying framework for understanding these works and provides a useful perspective toward the design of future experiments.

## Graphical abstract

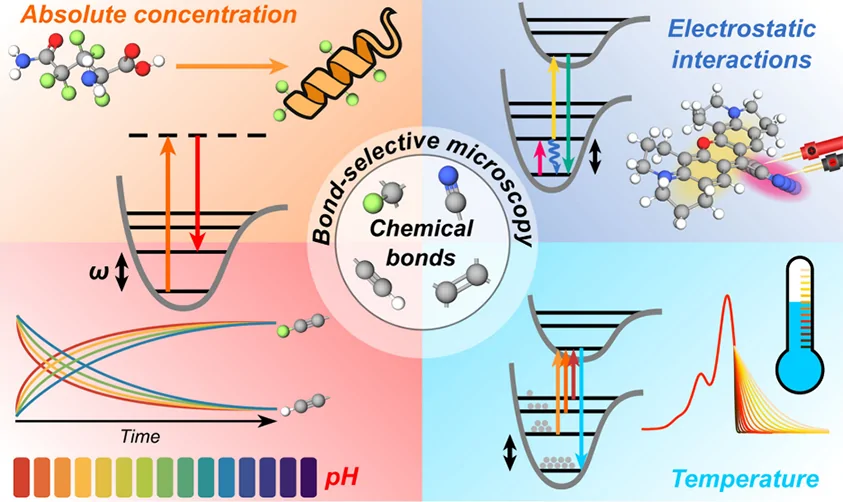

## Key References

Bi, X. , Suen, B. , Lin, L.-E. , Miao, K. , Wei, L. GFP-free live neuron quantitative imaging reveals compartmentalization and growth dynamics of polyQ aggregates. Proc. Natl. Acad. Sci. U.S.A. 2026, 123, e2505321123.10.1073/pnas.2505321123PMC1284969241564128 ^1^ This paper extends our previous fluorescence-free protein aggregate imaging into live neurons, where quantification in absolute concentrations is achieved with stimulated Raman scattering microscopy and reveals heterogeneous aggregate structures.

Bi, X. , Miao, K. , Wei, L. Alkyne-Tagged Raman Probes for Local Environmental Sensing by Hydrogen–Deuterium Exchange. J. Am. Chem. Soc. 2022, 144, 8504–8514.10.1021/jacs.2c0199135508077 ^2^ This paper demonstrates local pH (pD) sensing through hydrogen–deuterium exchange with terminal alkynes. Upon exchange, the alkyne stretching frequency exhibits a strong redshift, allowing for direct measurement with stimulated Raman scattering microscopy.

Kocheril, P. A. , Lee, D. , Naji, N. , Chadha, R. S. , Leighton, R. E. , Wang, H. , Wei, L. Single-Molecule Vibrational Thermometry. J. Phys. Chem. B 2025, 129, 8788–8797.10.1021/acs.jpcb.5c04024PMC1239651540814186 ^3^ This paper develops Boltzmann-edge vibrational thermometry as a calibration-free method of measuring temperature via the Boltzmann vibrational energy distribution. Employing anti-Stokes fluorescence detection, we demonstrate single-molecule thermometry and extend this concept to cell imaging and quantifying laser-induced heating.

Kocheril, P. A. , Wang, H. , Lee, D. , Naji, N. , Wei, L. Nitrile Vibrational Lifetimes as Probes of Local Electric Fields. J. Phys. Chem. Lett. 2024, 15, 5306–5314.10.1021/acs.jpclett.4c00597PMC1148645238722706 ^4^ This paper develops bond-selective fluorescence spectroscopy in the time domain. We show that nitrile vibrational lifetimes can act as reporters of local electric fields, providing both direct experimental measurements and first-principles calculations.

## 1 Introduction

Biological systems are inherently complex, with pronounced subcellular structure that is intimately tied to biochemical function. To maintain distinct molecular compositions in different organelles, cells must be able to regulate chemical reactivity with nanoscopic precision (Figure 1, left). In recent years, there has been a growing appreciation for the chemical complexity of this problem, with liquid–liquid phase separation being a notable mechanism by which cells can dictate local reactivity.^5,6^ Yet, the precise mechanisms by which cells achieve this local regulation remain poorly understood, particularly from a chemical perspective.^7^

**Figure 1:**
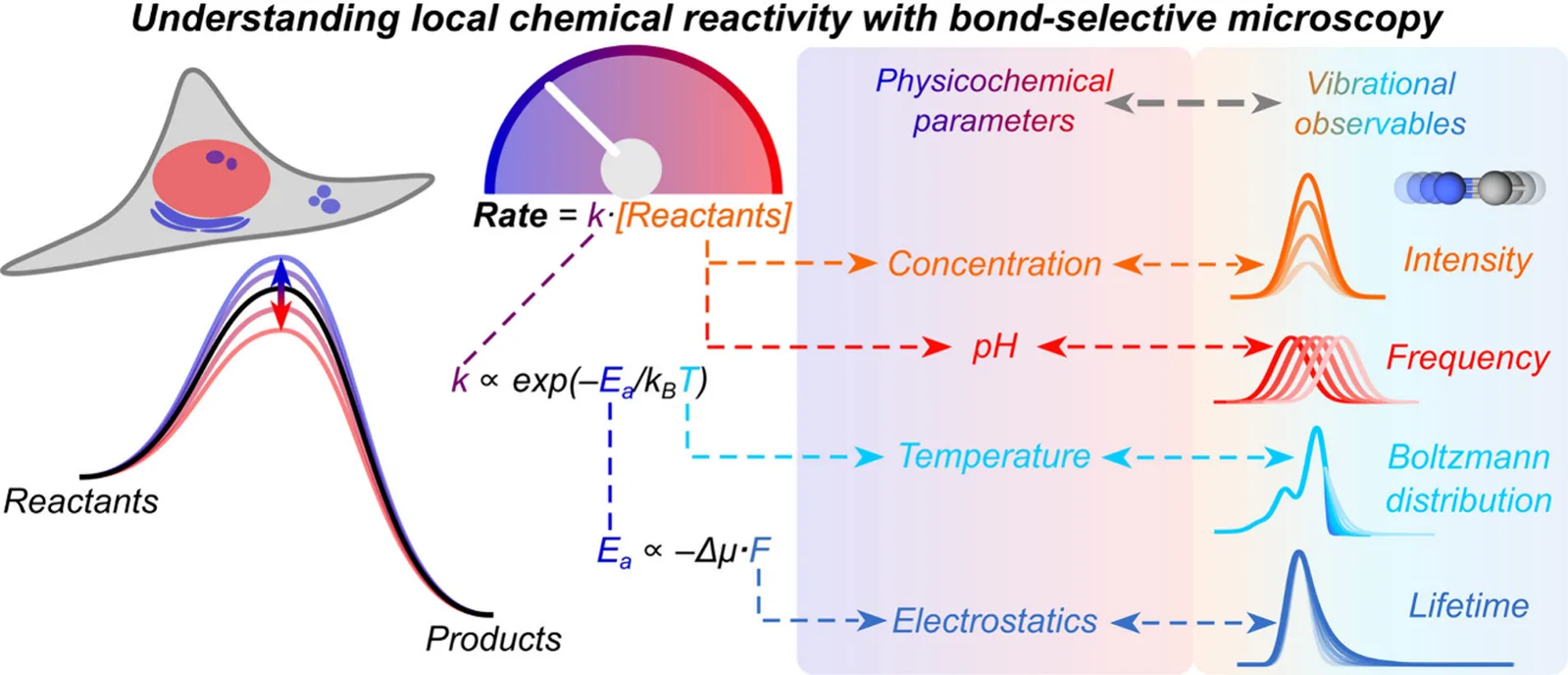
Bond-selective microscopy as a probe of chemical regulation. Reaction rates are governed by several physicochemical parameters, such as concentration, pH, temperature, and electrostatics, which can each be quantitatively measured with vibrational spectroscopic observables.

Chemical kinetics provides a comprehensive basis for quantitatively understanding the regulation of chemical reactions (Figure 1, center).^8^ From a handful of basic physicochemical parameters (reactant concentrations, pH, temperature, and local electrostatics), rate constants and reaction rates can be quantitatively modeled with high success,^9^ even extending to large-scale networks (e.g., stratospheric chemistry).^10^ The challenge in applying this framework to biochemical systems lies not in the scale of the networks, but rather in the measurement of the governing physicochemical parameters, with quantitative accuracy and high spatial resolution, in the native environment of a living cell.

Chemical reactions involve forming and breaking chemical bonds; molecular vibrations, derived from the stretching and bending motions of chemical bonds, thereby provide a natural context for directly measuring structural changes through changes in molecular vibrational “fingerprints.” Therefore, vibrational spectro-microscopies are highly attractive toward understanding local chemical organization in biological systems.^11^ As we detail in the following sections, vibrational spectroscopic observables (intensities, peak frequencies, energy distributions, and lifetimes) can be quantitatively linked to the physicochemical parameters of interest (Figure 1, right).

In this Account, we present our group’s work on developing functional bond-selective imaging strategies, featuring vibrational imaging platforms integrated with matching functional reporter molecules to quantitatively probe key physicochemical parameters. Grounded in fundamental principles, these approaches exploit vibrational intensity, frequency shifts, population changes, and relaxation kinetics to provide new avenues for uncovering hidden biological and chemical insights. Using stimulated Raman scattering (SRS) with stable isotope labeling, we quantify the concentration of mutant Huntingtin (mHtt) protein in cellular aggregates,^12^ observing multiple distinct aggregate phenotypes in neurons.^1^ Leveraging alkyne-tagged metabolites, we demonstrate that alkyne hydrogen–deuterium exchange kinetics offer a readout of local pD in live cells.^2^ We then discuss the development of two new techniques, termed Boltzmann-edge vibrational thermometry (BET)^3^ and bond-selective fluorescence-detected infrared-excited (BonFIRE) spectro-microscopy.^13^ With BET, we demonstrate that electronic absorption from thermally populated vibrational excited states, reflecting the Boltzmann distribution, allows for quantitative thermometry in cells and with single-molecule sensitivity.^3^ Finally, with BonFIRE spectro-microscopy, we observe that nitrile vibrational lifetimes are correlated with local electric fields and derive the mechanism governing this sensitivity.^4^ By developing functional methods for quantifying local cellular parameters, we hope to further our understanding of complex cellular processes and regulation of chemical activity.

## 2 Foundations of Functional Bond-Selective Imaging

Functional bond-selective imaging is built on the foundations of advanced spectro-microscopy methods,^14^ vibrational reporter design,^15^ and strategies for incorporating functional, bioorthogonal tags.^16^ Because these advances have been recently reviewed elsewhere,^14–17^ we will only briefly discuss these aspects here. Although there are notable exceptions,^18^ most vibrational imaging approaches leverage contrast from one of two physical processes: infrared (IR) absorption and Raman scattering. Despite having complementary selection rules, both are generally limited to mM sensitivity in conventional instrumentation with slow acquisition, precluding fast detection of low-abundance biomolecules.^14^

To overcome these sensitivity limitations, a variety of signal amplification mechanisms and clever detection schemes have enabled high-sensitivity, high-speed vibrational microscopy.^14^ While traditional (spontaneous) Raman scattering suffers from intrinsically weak cross-sections (∼10^–30^–10^–28^ cm^2^), SRS^19,20^ achieves up to ∼10^6^–10^8^-fold signal enhancement over spontaneous Raman scattering, leveraging the coherent signal amplification of stimulated emission.^21^ SRS maintains high spectral fidelity and exhibits a straightforward, linear concentration dependence, with detection limits in the 0.1–10 mM range;^16^ these favorable characteristics have made SRS a workhorse of modern vibrational imaging, allowing detailed studies of endogenous biomolecules without adding any exogenous labels.^22,23^ However, the spectra of endogenous chemical species are complex and highly overlapped, limiting molecular specificity, dynamic tracking, and quantitation.^16^ As we will discuss in the following sections, the introduction of bioorthogonal tags as vibrational spectroscopic probes grants additional functionality, including quantifying absolute concentrations in single protein aggregates in live cells^12^ and neurons^1^ and reporting local cellular pH.^2^

In contrast, IR absorption cross-sections can be quite large (∼10^–19^–10^–17^ cm^2^),^24^ but IR microscopies traditionally suffer from noisy detection and coarse spatial resolution while operating at the diffraction limit.^14,25^ These challenges have spurred the development of IR action spectroscopies, where another observable, such as thermal lensing or fluorescence, is used as an indirect measure of IR absorption.^26,27^ BET and BonFIRE are both fluorescence-detected action techniques, gaining improved (single-molecule) sensitivity, specificity, and spatial resolution over conventional IR microscopy. Coupled with engineered probe molecules, action microscopy allows for single-molecule IR imaging under ambient conditions,^28^ video-rate imaging on live cells,^29^ and, as we will discuss, quantitative measurements of local temperature^3^ and electric fields.^4^

## 3 Quantitative Imaging of Polyglutamine Aggregates in Absolute Concentrations

Concentration is perhaps the most basic chemical observable, indicating the local abundance of a given species (Figure 1). Dating back to the earliest work from our group, we hypothesized that measurements of absolute concentrations could be informative in neurodegenerative disease, where many mechanistic questions on the nature and formation of protein aggregates remain unanswered.^12^ Conventional biochemical approaches struggle to quantitatively measure concentrations in live cells, often requiring extraction and purification or sample fixation.^30–33^ Even fluorescence microscopy, the gold standard method for live-cell optical imaging, faces challenges in using intensity as a measure of concentration, due to photobleaching and the influence of electronic solvatochromism on fluorescent brightness.^4^ Moreover, fluorescent tags can be rather large, potentially perturbing biological organization, localization, and function.^1^

In a recent pair of studies, our group demonstrated that SRS microscopy combined with selective stable isotope labeling allows for detailed compositional profiling of protein aggregates formed by mutant Huntingtin exon 1 (mHtt-exon1) proteins in live HeLa cells and neuronal cocultures (Figure 2a).^1,12^ Huntington’s disease is a polyglutamine (polyQ) disease, characterized by long sequences (>40) of CAG trinucleotide repeats that produce elongated polyQ regions in mHtt proteins.^34–36^ These expanded polyQ domains strongly promote protein aggregation, yet the pathological role of these aggregates remains under debate.^30,31,37,38^ Leveraging the fact that mHtt-exon1, a key spliced peptide implicated in disease onset, is highly enriched in glutamine (∼68%) relative to the baseline incorporation in the human proteome (∼4%), we demonstrated that incubation with deuterated glutamine (Gln-d_5_) yields enrichment of C–D bonds in mHtt-exon1 aggregates.^12^ This approach yields multiple advantages: first, C–D stretches resonate in the cell-silent region (1800–2600 cm^–1^), where there is minimal vibrational background from endogenous biomolecules; second, small vibrational tags exhibit minimal biomolecular perturbation and are non-photobleaching;^15,39^ third, C–D Raman scattering cross sections are largely environment-invariant, enabling construction of a calibration curve that directly links SRS intensity to absolute concentration in solution (Figure 2b). This calibration can then be applied to live-cell measurements to obtain absolute quantification of polyQ aggregates *in situ* (Figure 2c) with an error of ± 0.4 mM.^12^

**Figure 2:**
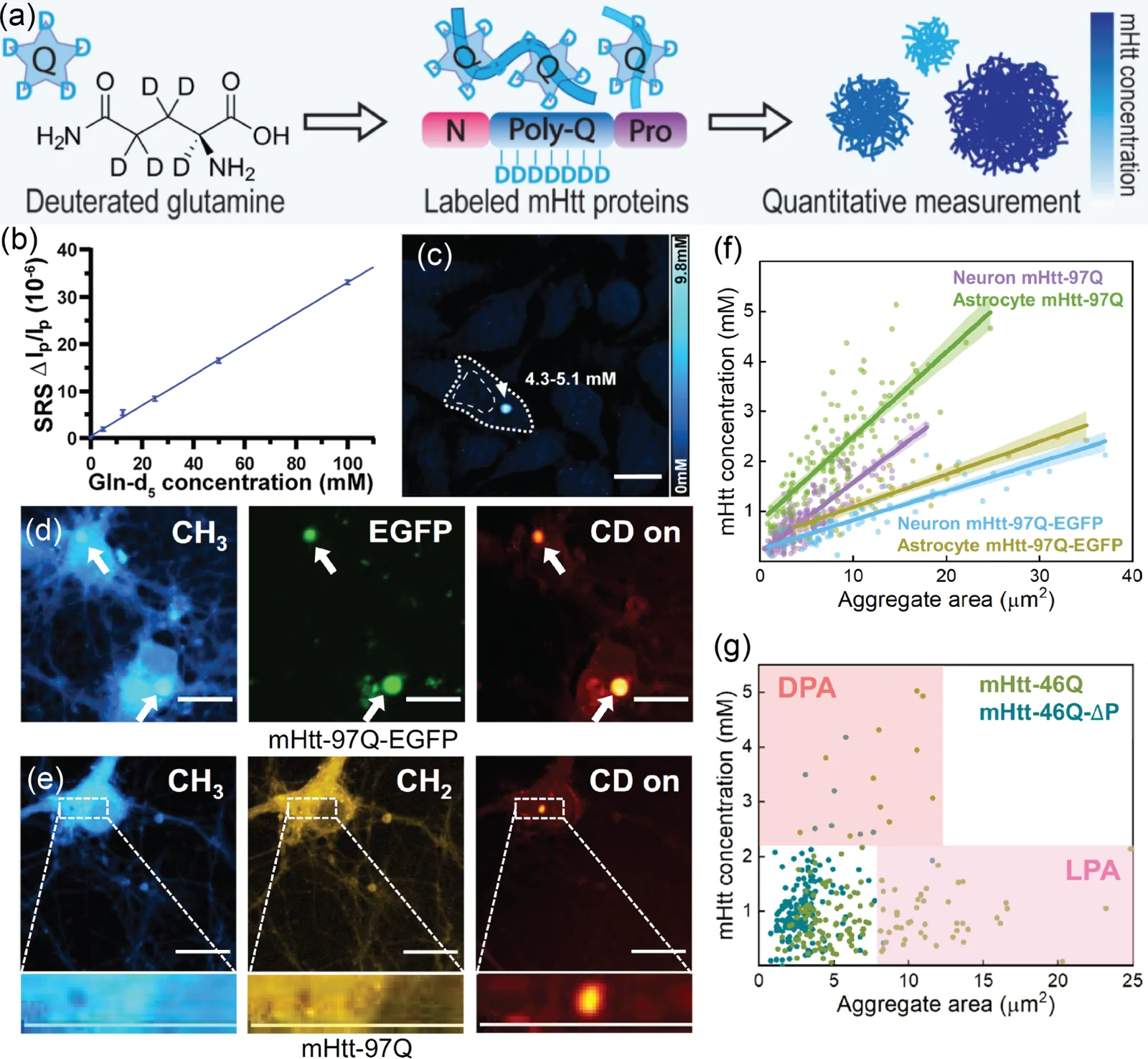
Quantitative analysis of mHtt aggregates through selective glutamine deuteration. (a) Scheme of Gln-d_5_ incorporation into mHtt-exon1 proteins, which then induce protein aggregation. (b) Linear dependence of C–D SRS signal intensity with Gln-d_5_ concentration. (c) C–D SRS image of a mHtt-97Q aggregate in a HeLa cell with its calculated mHtt concentration. Cell border and nucleus are outlined with dotted lines. (d, e) SRS and EGFP fluorescence images from the same neurons expressing mHtt-97Q-EGFP (d) or mHtt-97Q (e). (f) mHtt concentration as a function of aggregate size for mHtt-97Q and mHtt-97Q-EGFP in neurons and astrocytes. (g) Distribution of mHtt concentration as a function of aggregate size for mHtt-46Q (light green) and mHtt-46Q-ΔP (dark green). Two distinct populations are labeled as densely packed aggregates (DPA, coral-shaded) and loosely packed aggregates (LPA, pink-shaded). Scale bars: 20 μm. (Panels b and c adapted from ref 12. Copyright 2020 American Chemical Society. Other panels adapted with permission from ref 1. Copyright 2026 National Academy of Sciences.)

After demonstrating this concept in HeLa cells, we extended our approach to primary hippocampal neuronal cocultures with adeno-associated virus transduction to study aggregation in a more physiologically relevant, complex environment.^1^ We successfully imaged an enhanced green fluorescent protein (EGFP)-tagged mHtt-exon1 construct with 97 CAG repeats (mHtt-97Q-EGFP; Figure 2d) and an untagged version (mHtt-97Q; Figure 2e) in neurons. Consistent with our HeLa cell observations, untagged proteins formed denser aggregates compared to fluorescently tagged variants. Quantitative analysis further revealed that mHtt concentration increased with aggregate size in both neurons and astrocytes (Figure 2f), while non-mHtt protein concentration remained largely independent of aggregate size. Moreover, mHtt-97Q-EGFP aggregates exhibited lower mHtt concentration despite forming larger aggregates (Figure 2f), reinforcing the conclusion that fluorescent tags alter both aggregate composition and structure.

Having validated our quantitative framework, we then explored a series of experimental modifications, beginning with varying the polyQ repeat length (97Q vs 46Q) and deleting the C-terminal proline-rich region (ΔP) in the mHtt-exon1 sequence (Figure 2a, middle).^1^ These experiments yielded fascinating insights, revealing the existence of distinct subpopulations of polyQ aggregates in neuronal cocultures. For example, comparing mHtt-46Q and mHtt-46Q-ΔP aggregates, we observed clustering into two distinct groups, which we termed loosely packed aggregates (LPA) and densely packed aggregates (DPA), as a reflection of the mHtt concentration relative to the aggregate size (Figure 2g). Examining aggregate morphologies, we saw that LPAs were appreciably more elliptical than DPAs and that the ΔP deletion tended to result in DPAs for both the 97Q and 46Q constructs. Moreover, by integrating neuron-specific staining to differentiate between neurons and astrocytes, we observed cell type-dependent differences in aggregate subcellular localization, morphology, and density. These experiments convincingly demonstrate that, by developing an imaging technique capable of reporting absolute concentration while remaining minimally perturbative, we gained significant insights into neuronal biology, discovering substantial subcellular heterogeneity in polyQ aggregates in neurons. Moreover, these works establish the power of C–D Raman probes for absolute concentration determination in live cells; the extension of this concept to probing metabolic reaction networks is currently an active area of work for our group.^40^

## 4 Quantitative Intracellular pD Sensing by Alkyne Hydrogen–Deuterium Exchange

Local pH dictates the protonation state (and thereby, reactivity) of chemical species (Figure 1), which is particularly important in the context of carbonyl and enol­(ate) chemistry, where many reactions are acid- or base-catalyzed. Biochemically, regulation of local pH is crucial for enzyme activity, where the protonation states of key residues in active sites (e.g., aspartate and histidine) are intimately tied to biological function.^41^ As a result, pH dysregulation is a hallmark of numerous pathological conditions, including cancer,^42–46^ chronic inflammation,^47^ and lysosomal storage disorders.^47–49^ The ability to resolve subcellular pH gradients would provide a valuable window into biological function, but quantitative pH imaging remains challenging. Many fluorescent dye-based “turn-on” and ratiometric pH sensors have been reported, but intensity-based readouts can be confounded by photobleaching, uneven probe distribution, and dye leakage, compromising accuracy.^50^ Genetically encoded fluorescence lifetime-based reporters address many of these issues,^51^ but the introduction of a fluorescent tag can be quite perturbative (as we saw with polyQ aggregates). Moreover, fluorescence-based sensors rely almost entirely on empirical observation and standardization with experimental controls. In contrast, we believe that sensors with physically grounded, rationally interpretable sensing mechanisms may provide complementary, and even deeper quantitative insights and should be less susceptible to confounding variables, such as viscosity, temperature, and the local presence of quenchers (such as water).^52–54^ In this context, vibrational spectroscopy, with deep connections between first-principles theory and experiments, presents a unique opportunity for quantitative, physically interpretable local pH sensing.

To design a vibrational pH sensor, we drew inspiration from hydrogen–deuterium exchange (HDX) as practiced in nuclear magnetic resonance spectroscopy^55,56^ and mass spectrometry,^57–60^ where backbone amide protons exchange with deuterated solvents to reveal protein secondary and tertiary structure. We hypothesized that terminal alkynes, which possess comparable p*K*_a_ values, could similarly undergo HDX from C≡C–H to C≡C–D (Figure 3a).^2^ Where Raman pH sensors commonly involve larger exogenous probes or empirical peak ratios,^61,62^ we were optimistic that alkyne HDX could preserve strong physical interpretability while remaining minimally perturbative. Alkynes are particularly attractive targets in this context, since alkyne-tagged metabolites can be metabolically incorporated into biomacromolecules and exhibit strong Raman scattering signals in the cell-silent region.^63–65^

**Figure 3:**
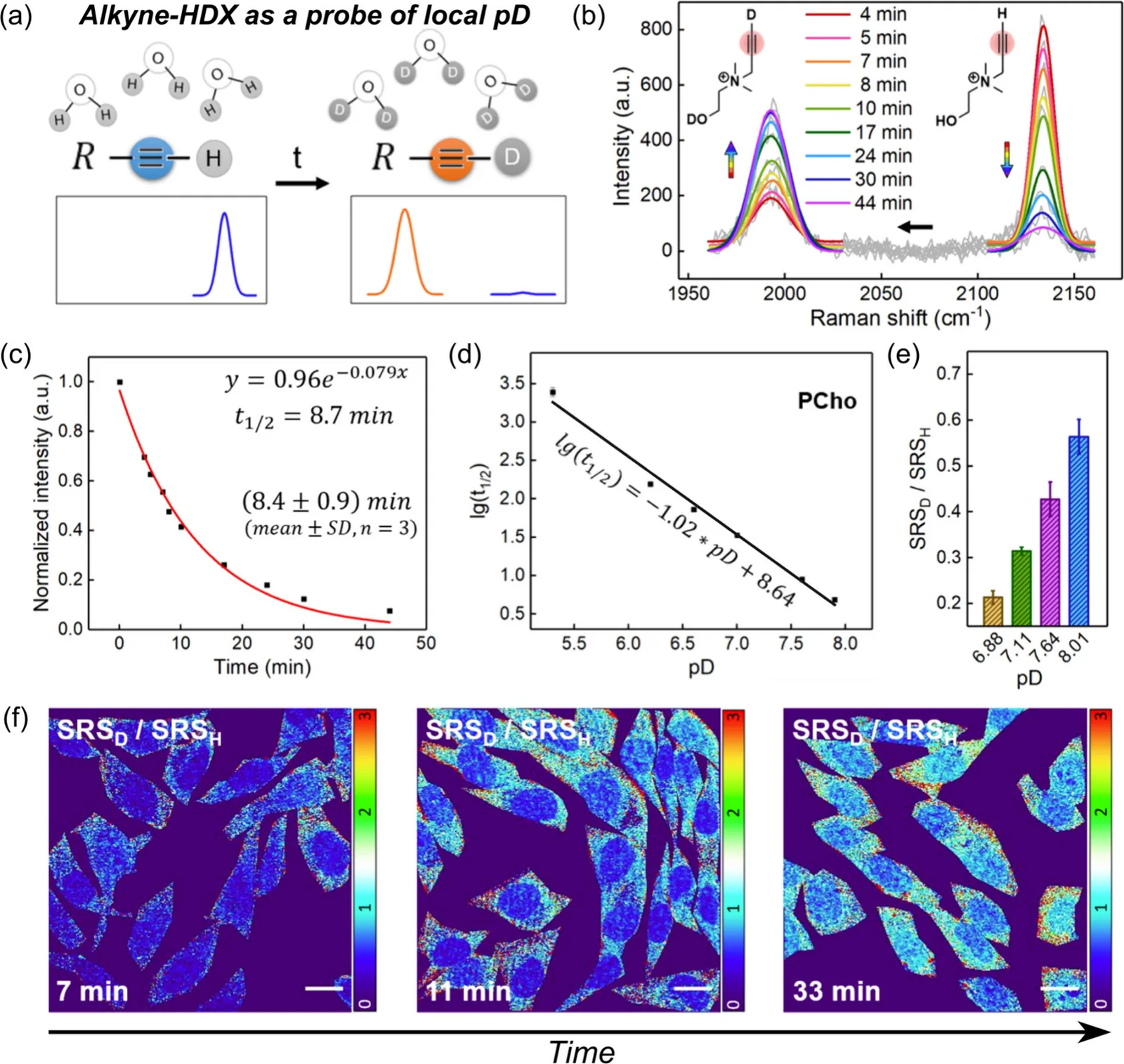
Alkyne-HDX platform for measuring and mapping intracellular pD. (a) Concept of alkyne-HDX-based Raman sensing. (b) Spontaneous Raman spectra of PCho during alkyne-HDX. (c) Exponential fitting of C≡C–H peak intensities for PCho over time. (d) Linear relationship between lg­(*t*_1/2_) and pD for PCho. (e) Intracellular pD changes upon treatment with nigericin, reported by SRS_D_/SRS_H_ of PCho in live cells. (f) Ratiometric imaging of EdU-labeled DNA and PCho-labeled membrane with distinct alkyne-HDX rates. Scale bars: 20 μm. (Adapted from ref 2. Copyright 2022 American Chemical Society.)

Upon immersion in D_2_O, we observed that propargylcholine (PCho), a model alkyne-tagged metabolite, readily underwent HDX, evidenced by a gradual decrease in C≡C–H alkyne-stretching Raman intensity and a corresponding increase in C≡C–D alkyne-stretching Raman intensity (red-shifted by ∼130 cm^–1^) over time (Figure 3b).^2^ We confirmed that several other alkynes exhibited similar responses, establishing the generality of alkyne-HDX with significant potential for reporting local pH. Following the framework of amide-HDX in proteins, we modeled alkyne-HDX as a base-catalyzed, pseudo-first-order reaction in D_2_O. We derived that the logarithm of the exchange half-life (*t*_1/2_) scales linearly with both the alkyne p*K*_a_ and the surrounding pD (lg­(*t*_1/2_) = p*K*_a_ – pD + constant), where more acidic alkynes and more basic conditions lead to faster exchange. As an example, an alkyne with a p*K*_a_ of 20 was calculated to have a *t*_1/2_ of 174 s. In excellent agreement with our theoretical model, we observed single-exponential kinetics (Figure 3c) and a robust linear relationship between lg­(*t*_1/2_) and pD (Figure 3d), indicating that for a given alkyne probe, measuring lg­(*t*_1/2_) *in situ* provides a quantitative readout of the local pD with an error of ±5%.

We next sought to develop alkyne-HDX as a live-cell imaging modality with SRS, toward spatially mapping intracellular pD gradients.^2^ We first treated cells with nigericin, an ionophore that overcomes the cell’s intrinsic buffering capacity and allows modulation of the extracellular buffer pD to set the intracellular pD. For fast, live-cell imaging, we imaged the SRS C≡C–D/C≡C–H ratio (SRS_D_/SRS_H_, as a reflection of the HDX kinetics at a single time point), observing sensitive tuning by more than 2-fold across an external pD range spanning less than one pD unit (Figure 3e), with an ∼40% larger fold-change over the same pD range than a traditional, ratiometric fluorescence sensor.^2^

We then exploited the p*K*_a_-sensitivity of alkyne-HDX, where different probes exhibit different exchange kinetics under the same buffering conditions.^2^ By coincubating 5-ethynyl-2’-deoxyuridine (EdU; a slow-exchanging, nuclear probe) and PCho (a fast-exchanging, cytoplasmic probe), we observed that nuclear regions consistently displayed much lower SRS_D_/SRS_H_ ratios than cytoplasmic membrane regions across multiple time points after D_2_O buffer incubation (Figure 3f). This demonstrates that kinetic contrast, rather than spectral contrast, can serve as an additional imaging dimension for multiplexing alkyne-tagged probes that could otherwise be difficult to distinguish due to overlapping vibrational frequencies. Although further work is needed to fully understand these results, these experiments again demonstrate that the development of a quantitative vibrational imaging platform and its application to complex biological systems is a fruitful framework for discovering novel, physically informed insights in chemistry and biology. Furthermore, alkyne probes are particularly attractive as pH sensors in crowded environments that are inaccessible to larger fluorophores, such as enzyme active sites; we envision several applications using novel alkyne-labeled molecules that can shed light on the role of local pH in modulating intracellular reaction rates, such as alkyne-labeled sugar analogues for probing pH regulation of sugar chemistry.^66^

## 5 Calibration-Free Local Vibrational Thermometry with Single-Molecule Sensitivity

Temperature is yet another central physicochemical variable that governs reactivity in chemical and biological systems.^52^ In chemical systems, increasing temperature raises molecular thermal energy and thus reaction rates (Figure 1). In biological systems, the same principle applies, but the outcome is further shaped by the temperature dependence of enzymatic activity. Because reactivity is determined by the temperature at local reaction sites rather than by the average bulk temperature, accurate local thermometry can provide deeper mechanistic understanding by enabling temperature to be measured as a spatially resolved variable.^67^

A well-established method for measuring local temperature is fluorescence-based molecular thermometry; fluorescence thermometers are generally based on thermal quenching, where elevated temperatures reduce the fluorescence lifetime and brightness.^68–70^ Fluorescence thermometry provides thermal maps that can reveal how biological processes vary spatially. Such measurements are typically noninvasive and highly sensitive, enabling detection of small temperature changes in specific subcellular locations. However, fluorescence-based thermometers exhibit relatively small dynamic ranges (∼50 K) and generally do not reveal the molecular sources responsible for the observed heating, in addition to the above-mentioned confounds of fluorescence sensors.^52–54^ These limitations have motivated the search for thermometry methods with a more direct, physical basis.

Toward this goal, our group developed Boltzmann-edge vibrational thermometry (BET) for quantitative local thermometry (Figure 4a).^3^ The essential idea is that the signal intensity in the long-wavelength tail of a UV–vis absorption spectrum, known as the Boltzmann edge, reflects the population of vibrational states occupied at thermal equilibrium (Figure 4b). Because these populations follow the Boltzmann distribution, the (exponential) energy dependence of the measured signal directly encodes temperature (Figure 4c).^3,71^ In this way, BET differs from conventional fluorescence thermometry because its temperature dependence arises from a defined thermodynamic population distribution rather than from an empirical probe response. Compared with other vibrational thermometry approaches, BET also offers substantially higher sensitivity by leveraging fluorescence detection.^72^ Moreover, BET is calibration-free and largely immune to local environmental variations because the relevant information is encoded in relative rather than absolute intensity. As a result, BET is especially attractive as a quantitative, molecule-resolved thermometric strategy for chemically and spatially heterogeneous systems.

**Figure 4:**
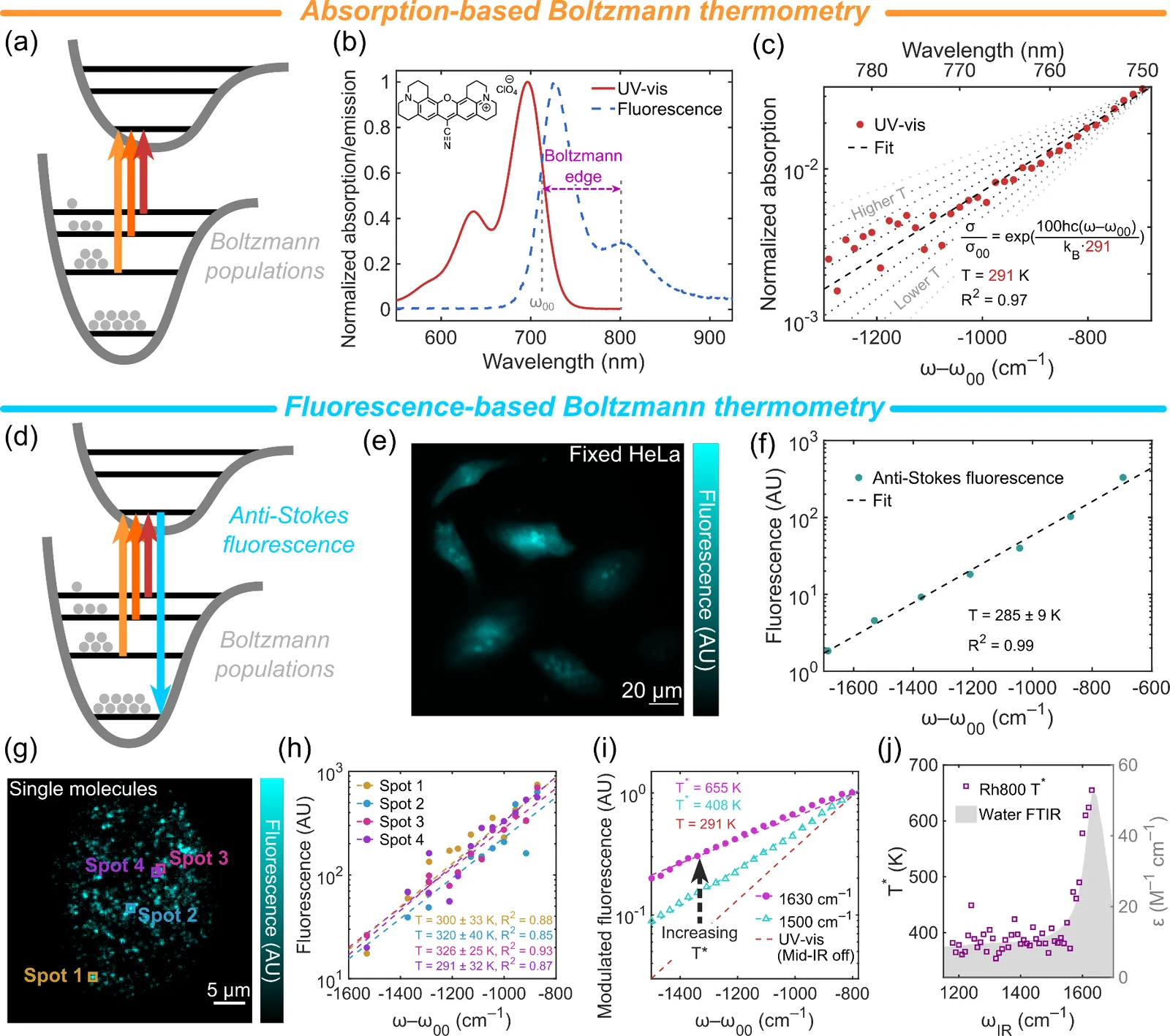
Boltzmann-edge vibrational thermometry. (a) Concept of Boltzmann-edge absorption, as a reflection of the Boltzmann populations in vibrational excited states. (b) Normalized absorption and emission spectra of Rh800 in DMSO at room temperature (structure inset). (c) Log-scale view of the Boltzmann edge in the UV–vis absorption spectrum; the dashed line corresponds to 291 K, and dotted lines illustrate shifts with temperature. (d) Concept of anti-Stokes fluorescence-based Boltzmann thermometry. (e) Anti-Stokes fluorescence image of Rh800-stained HeLa cells. (f) Boltzmann-edge analysis of anti-Stokes fluorescence intensity, yielding a mean cellular temperature of 285 ± 9 K (95% confidence). (g) Anti-Stokes fluorescence image of Rh800 molecules in a poly­(vinyl alcohol) thin film with four spots indicated. (h) Boltzmann-edge measurements from individual molecules, giving an ensemble mean temperature of 289 ± 19 K (95% confidence). (i) BET measurements under mid-IR irradiation, leading to local heating. (j) BET fingerprinting, demonstrating that local vibrational temperatures can be used to identify the molecular origin of local heating. (Adapted from ref 3. Copyright 2025 American Chemical Society.)

We then hypothesized that anti-Stokes fluorescence, resulting from excitation of thermal populations in vibrational excited states, could allow for sensitive BET microscopy (Figure 4d).^3^ By staining fixed HeLa cells with Rhodamine 800 (Rh800), a model near-IR dye, we observed bright anti-Stokes fluorescence (Figure 4e), which decayed exponentially as we tuned our excitation laser wavelength, allowing us to map the Boltzmann edge in a biological context (Figure 4f). The reported temperature of 285 ± 9 K suggests that the cells remained near ambient conditions, with no appreciable heating from the near-IR illumination. More broadly, this shows that BET is not restricted to simplified model systems but can return reasonable temperatures in biologically relevant settings.

Encouraged by our bioimaging results, we pushed BET into even more demanding regimes.^3^ At the single-molecule level, the appearance of isolated bright spots indicates highly localized emitters (Figure 4g), while the accompanying fluorescence decrease can be interpreted within the same Boltzmann-edge framework used for ensemble measurements (Figure 4h). From this analysis, we measure a mean temperature of 289 ± 19 K from single molecules. Although the uncertainty is naturally larger in this regime because of the reduced photon budget, the measured value remains physically reasonable and consistent with ambient conditions. In parallel, upon coillumination with a mid-IR laser in solution, we observed remarkable shifts in the Boltzmann edge (Figure 4i). Because BET is a direct readout of vibrational populations, these measurements indicate significant vibrational excited populations, comparable to temperatures exceeding several hundred K. These measurements illustrate that vibrational temperature (T*), reflecting the Boltzmann *vibrational* energy distribution, can differ from *translational* temperature. Under steady-state conditions (Figure 4a–h), the two are the same, and thus vibrational temperature can be used as a readout of translational temperature. However, vibration-translation energy exchange generally occurs on relatively slow (μs–ms) time scales, meaning that measurements with ps time resolution (Figure 4i–j) can probe time points when vibrational temperature and translational temperature are not yet well-equilibrated (e.g., under laser heating on ultrafast time scales).^73^

Interestingly, such elevated vibrational temperatures have been predicted to exist on ultrafast time scales due to the presence of bottlenecks to energy transfer, resulting from vibrational heating within the focal spot.^74^ By mapping T* as a function of the mid-IR frequency, we observed a peak at ∼1630 cm^–1^, overlapping with the IR extinction coefficient of water (Figure 4j). We termed this approach “BET fingerprinting”, where the profile of T* allowed us to identify the molecule responsible for the observed laser-induced heating. We envision that BET fingerprinting may be best applied to quantitatively tracking local heat generation and propagation for applications like mid-IR neuromodulation.^75^

Taken together, these examples highlight the broader promise of Boltzmann vibrational population mapping for quantitative local thermometry. BET is especially compelling in this regard because it combines physically grounded thermometry with chemical specificity, applicability in biological settings, and single-molecule sensitivity. Moreover, by developing BET fingerprinting, we observed transient temperatures on ultrafast time scales, providing new insights into energy flow and bottlenecks following laser heating. BET exemplifies how vibrational imaging in the pursuit of quantitative physicochemical reporting can spur the discovery of fundamental chemical knowledge. Looking forward, we are optimistic about the potential for BET to probe subcellular thermal regulation in living cells, which is an area of active debate,^76,77^ and shed light on regulation of chemical activity. Similarly, we hope that BET fingerprinting can provide a quantitative basis for new biological discoveries related to thermal conductivity and transport, particularly in mid-IR neuromodulation.

## 6 Nitrile Vibrational Lifetimes as Electrostatic Reporters

Intermolecular interactions are the major drivers of many biological phenomena, including DNA base-pairing, enzymatic activity, and more.^78^ Toward a unifying framework, Boxer and co-workers have convincingly argued that the local electric field provides a uniquely quantitative basis for describing these diverse noncovalent interactions.^79^ Spectroscopically, local electric fields can be measured through Stark effects, or electric field-induced spectral changes,^80,81^ with local oscillators like nitriles and carbonyls being particularly well-suited to this task.^79,82,83^ While many fluorescence-based electric field reporters have been developed and used for a variety of imaging applications, these probes require stringent standardization and extensive experimental controls.^84,85^ In contrast, the power of the vibrational Stark effect lies in its strong physical underpinnings, fostering broad generalizability; as such, several groups have advanced IR spectroscopy for studying biological molecules in solution, gaining deep insights into structure, function, and dynamics.^86–90^

Inspired by this body of work, we sought to bring such rich, quantitative chemical insights to the imaging of living cells. Among the many technical developments reported to enhance sensitivity, specificity, and spatial resolution in IR imaging, we noted a particularly promising spectroscopy experiment reported by Laubereau, Seilmeier, and Kaiser (Figure 5a).^91^ They designed a two-pulse experiment, where the first (mid-IR) pulse excites a vibrational mode in a dye molecule, and the second (near-IR) pulse up-converts the vibrationally excited population to an electronic excited state, from which fluorescent emission can occur. The crucial detail is that the total fluorescent intensity is proportional to the IR absorption, allowing ultrasensitive action detection. Integrating modern instrumentation with narrow-band, high-repetition rate lasers and single-photon counting detection with an imaging-optimized near-IR dye palette, our group reported bond-selective fluorescence-detected IR-excited (BonFIRE) spectro-microscopy in 2023, demonstrating single-molecule sensitivity, multiplex imaging capability, and high biocompatibility (Figure 5a).^13^ Excitingly, several groups have reported similar strategies for a variety of applications in the chemical and biological sciences, illustrating the broad potential of this technique.^92–97^

**Figure 5:**
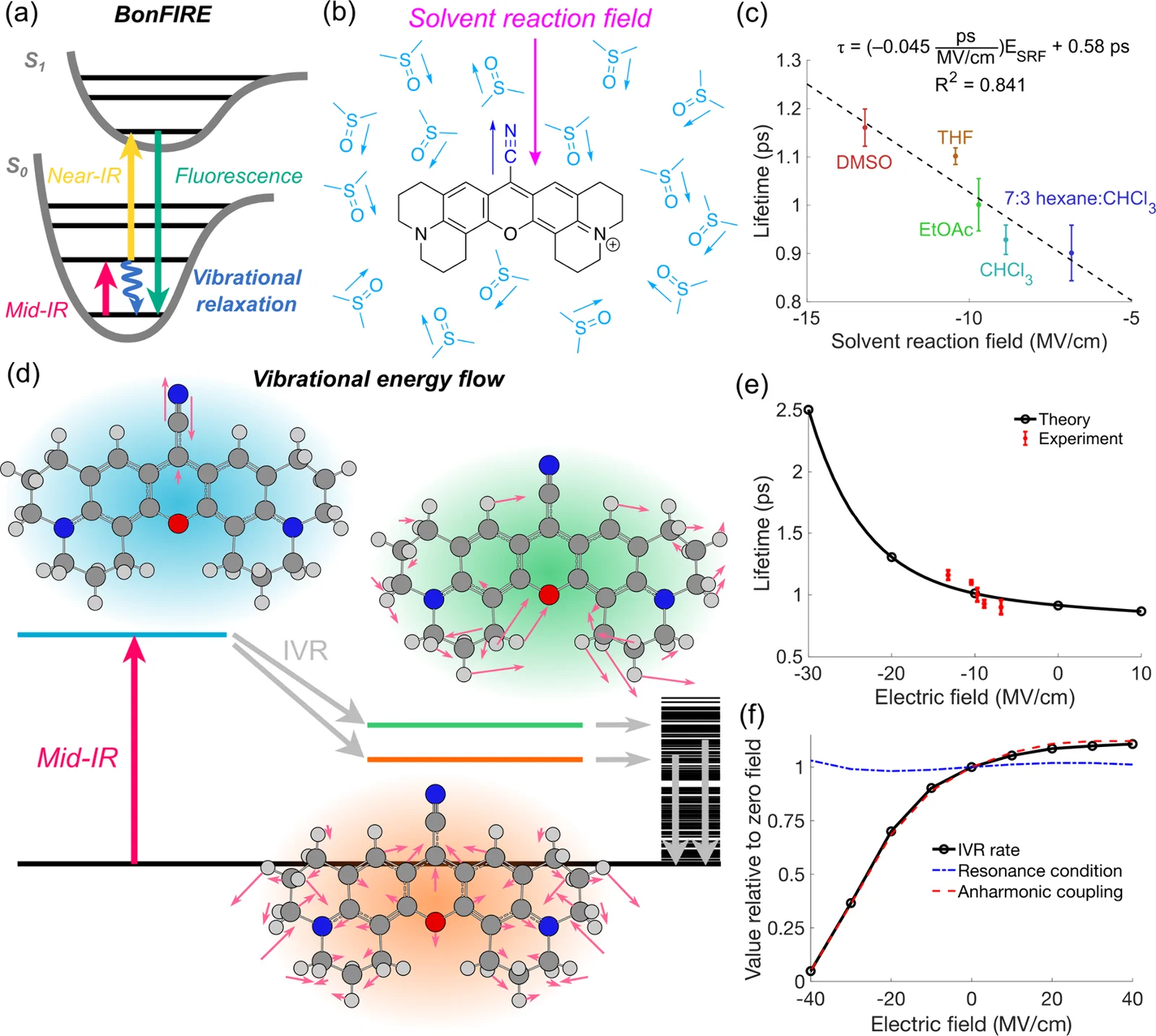
Nitrile vibrational lifetimes as probes of local electric fields. (a) Principle of BonFIRE spectroscopy. Vibrational lifetimes can be measured directly from fluorescent intensity by tuning the time delay between the mid-IR and near-IR pulses. (b) Structure of Rh800 and concept of a solvent reaction field. (c) Vibrational lifetime solvatochromism of the nitrile stretch of Rh800. (d) Doorway model of IVR, where energy from the nitrile-stretch of Rh800 is converted into two lower-energy modes before being dissipated in the dense bath of states. (e) Comparison between theoretical and experimental Rh800 nitrile lifetimes in varying electric fields. (f) Decomposition of the IVR rate, revealing changes in the anharmonic coupling as the dominant driver of Rh800 lifetime alterations. (Adapted from ref 4. Copyright 2024 American Chemical Society.)

Because we employ ultrashort (∼2 ps) pulses, BonFIRE can directly measure vibrational lifetimes; this is achieved by delaying the arrival of the near-IR pulse relative to the mid-IR. As the mid-IR-excited vibrational population relaxes, the population of molecules excited by the near-IR pulse decays proportionally, resulting in a decrease in overall fluorescent intensity. In the ultrafast spectroscopy community, vibrational lifetimes are known to provide dynamical information, such as local hydration or conformational changes;^98,99^ as such, we wondered about the potential for lifetime measurements with BonFIRE to provide access to new chemical information.

To explore this possibility, we examined the vibrational lifetime solvatochromism of the nitrile-stretching mode of Rh800 (Figure 5b).^4^ Solvatochromism provides a convenient means of assessing local electrostatic sensitivity, since solvents of differing polarity exert different “reaction” electric fields on dissolved solutes.^100,101^ Surprisingly, we observed a clear dependence of the nitrile lifetime of Rh800 in different solvents, which was quite linear with the calculated solvent reaction field (Figure 5c).^4^ The lifetime differences are subtle, but the high signal-to-noise ratio of BonFIRE enabled measurements with an average uncertainty of ±0.04 ps (∼1 MV/cm).

Given that vibrational lifetimes were traditionally considered molecular constants and not subject to change,^102^ we sought a mechanistic explanation for this intriguing trend. In large molecules, vibrational modes are depopulated by intramolecular vibrational energy redistribution (IVR), or anharmonic conversion into other vibrational modes (Figure 5d).^73,103^ Much of the fundamental theory for IVR was described in the latter half of the 20th century,^104–109^ but the framework described by Leitner and co-workers has appreciably bridged the gap between theory and experiments, allowing quantitative comparisons that yield deep insights into the underlying factors governing IVR.^110–112^

We thus performed anharmonic density functional theory (DFT) calculations in applied electric fields and calculated the corresponding nitrile vibrational lifetimes, obtaining robust agreement with our experimental data and observing increasing lifetimes with larger electric fields (Figure 5e). We then examined the field-dependence of the two primary contributions to the calculated IVR rate: the anharmonic coupling and the resonance condition (Figure 5f). The resonance condition, describing how well the vibrational decay conserves energy, had been the dominant factor in the IVR rates of smaller nitriles (benzonitrile and cyanophenylalanine),^112^ but it exhibited almost no variation for Rh800 (Figure 5f). Instead, the dominant contribution for Rh800 was the field-dependence of the anharmonic coupling (Figure 5f), reflecting equilibrium geometry alterations in response to the applied field.^110,113^

These results were particularly interesting, revealing that from the perspective of vibrational dynamics, Rh800 occupies a different molecular regime than smaller, prototypical nitrile probes like benzonitrile and cyanophenylalanine.^112^ By developing BonFIRE as an ultrasensitive IR spectro-microscopy capable of reporting local electric fields, we thus gained new insights into molecular phenomena, demonstrating the importance of the anharmonic coupling in vibrational decays in large molecules. Moreover, with recent developments of BonFIRE demonstrating single-molecule, wide-field imaging^28,29^ and super-multiplex imaging,^103^ we are excited about the potential for BonFIRE to provide new biological insights, and we are actively developing vibrational lifetime imaging and other strategies toward this goal. BonFIRE could thus allow direct assessment of subcellular spatial heterogeneity of enzymatic activity in live cells and its impact on overall cellular function.^17^

## 7 Outlook

In this Account, we have highlighted a handful of recent works from our group toward quantitative measurements of physicochemical parameters (absolute concentration, pD, temperature, and electric fields) in biological imaging. Vibrational spectroscopic observables are well-suited to reporting these parameters, enabling quantitative, *in situ* measurements in complex environments. Furthermore, we view the development of these methods as a means of discovery, providing new biological insights into polyglutamine aggregate heterogeneity and the influence of chemical structure on HDX kinetics, as well as chemical insights into vibrational energy flow and its sensitivity to molecular structure in live-cell contexts.

Overall, we view our methods as complementary to existing technologies, such as fluorescence imaging and electrochemical sensing,^114^ where vibrational spectroscopic observables are grounded in strong physical principles (reducing the possibility of bias from confounding variables) and provide a common quantitative language for interrogating many diverse physicochemical parameters in live biosystems (Figure 1). Moreover, the potential to simultaneously measure concentration, pH, temperature, and electrostatics is a unique possibility of these vibrational imaging approaches, which feature highly cross-compatible sample preparation and are nondestructive, and we are actively pursuing optical strategies (such as backscattered SRS detection) toward simultaneous measurements on real samples. Integrated measurements of all of these physicochemical observables could provide a fascinating, mechanistically detailed view of how chemistry happens in cells: correlative measurements of metabolite concentrations and local pH in the mitochondria could inform on mitochondrial function, or simultaneous measurements of local electrostatics and temperature could shed light on enzymatic activation mechanisms, or the formation of biomolecular condensates. Furthermore, deviations in such characteristic features could provide new insight into disease (e.g., uncoupling of metabolite concentrations and pH as an indicator of mitochondrial dysfunction), allowing us to start to understand the chemical mechanisms underlying biological and physiological dysregulation at the scale of chemical bonding.

We also expect that these methods should be generalizable beyond cultured cells, as both SRS and BonFIRE have previously demonstrated compatibility with more complex samples like tissues.^13,19^ While penetration depth remains a limitation for optical imaging, SRS, mid-IR photothermal, and fluorescence microscopy have all been demonstrated simultaneously on model organisms such as *C. elegans*.^115^ Toward these goals, another important challenge lies in the speed at which we can quantify the desired physicochemical parameters. Currently, our methods have acquisition times on the order of minutes, which is limited by the requirement to acquire multiple image frames at either differing frequencies (as with SRS and BET; ∼1–2 min tuning time with our current instrumentation) or optical time delays (for BonFIRE lifetime measurements; ∼200 ms settling time). We are optimistic that new technological innovations, such as faster-tuning laser instrumentation^116^ and echelon-based detection,^117^ may allow for faster imaging approaching real-time readouts.

Beyond bioimaging, the demands of high molecular specificity and sensitivity also make these methods attractive for other chemical fields of study, such as selective photochemistry,^118^ reactions at electrochemical interfaces,^119^ and single-molecule catalysis.^120^ We hope to see broader adoption of emerging vibrational spectro-microscopy methods throughout the chemistry community in the coming years, and given the relative simplicity of these techniques, we expect that optical spectroscopy groups should be able to implement similar methodologies. Toward broader adoption, we see a clear need for commercialization and development of a multimodal imaging platform capable of SRS, BonFIRE, and BET, similar to emerging integrated platforms capable of coherent Raman scattering, second-harmonic generation, and multiphoton fluorescence imaging.^121^ We also see a growing role for computations and modeling to complement vibrational spectroscopy and imaging.^122^ For example, extending our quantitative SRS framework to downstream metabolic products is enabled by metabolic reaction network mapping and network-scale DFT calculations, allowing for quantification of global cellular metabolism in absolute concentrations.^40^ Deeper integrations of experiments and theory should significantly advance our understanding of local biological environments from a chemical perspective and hold potential to shape the next decade of chemical imaging.
